# Alexandrite-like effect in purple flowers analyzed with newly devised round RGB diagram

**DOI:** 10.1038/srep29630

**Published:** 2016-07-11

**Authors:** Ichiro Kasajima

**Affiliations:** 1 Department of Agriculture, Iwate University, Ueda 3-18-8, Morioka, Japan

## Abstract

The gemstone alexandrite is known for its feature to change color depending on the spectral quality of the incident light. Thus, the stone looks green when illuminated by white LED light but looks red when illuminated by incandescent light. This effect (alexandrite effect) is caused by a special relationship between the spectral quality of the incident light and the absorbance spectrum of the stone. Here we report an alexandrite-like effect in the petals of torenia and cyclamen flowers. These flowers are purple in sunlight but magenta (reddish) in incandescent light, and violet (bluish purple) in white LED light. The *m-n*, *triangle* and *round* diagrams are devised to calculate the colors of visible light spectra, based on the RGB color-matching function. Using these calculations, the alexandrite-like effect in purple flowers was successfully analyzed in terms of the interaction between the incident light spectrum and the absorbance spectrum of their purple anthocyanin. This analysis allows both logical and intuitive understanding of the colors exhibited by any object showing alexandrite–like properties.

Gemstone alexandrite, found in Russia in 1830, is famous for having different colors under different incident lights. Alexandrite is green in white fluorescent light, whereas it is red in incandescent light. White, Roy and Crichton proposed the phrase ‘alexandrite effect’ to describe this phenomenon in 1967. Alexandrite effect is thought to have been observed between sunlight and candle-light in old times. Color of alexandrite is caused by chromium ion placed under a special chemical environment. Alexandrite effect is also a result of psycho-physical reaction due to the response of the human eye and brain, to particular part of the visible light spectrum^1^,^2^. Alexandrite effect is observed in some synthetic gemstones as well, but any additional natural material or organism showing such phenomenon would have been missing for nearly half century since this phrase was proposed.

For ornamental flowers, color and especially hue (white, red, yellow, blue, green, purple) is one of the key characters identifying cultivar, increasing variation and attracting consumers. For example, a common aim of ornamental flower breeders is the creation of novel petal colors^3^,^4^. The breeding of bluish flowers is more important than red because there are only a limited number of horticultural species with blue flowers. If we want to enhance the visual charms of flowers through color, it is important to better understand the mechanisms which generate flowers appearing blue to the human eye. So, not only the enzyme systems that synthesize the blue pigments, but also the physics of how the human eye senses the blue color is important. This study focuses on the interaction between pigments and the spectral quality of the light source - this sometimes makes purple flowers look blue.

Readers may have noticed that the color of purple flowers vary depending on the light source. For example, purple petunias look blue-purple under a dark sky after sunset, and purple cyclamens look red-purple at the window in daytime. Thus purple anthocyanin pigment in flowers seems to respond to the spectral quality of the incident light and changes its color. This response will be a special characteristic of purple anthocyanin, because such clear alteration of color is not observed in yellow or red flowers. This line of observation was already reported. Yang *et al*.^5^ compared flower colors under fluorescent lamps. Differences in petal hue measured by direct comparison with a Munsell color chart under 2,800 K warm-colored fluorescent lamp or a 6,500 K white LED light, were less than 15° for red, orange, yellow, white and yellow-green flowers. On the other hand, the differences in petal hue of blue-purple flowers were 42° and those of purple flowers were as high as 58° in the same set of measurements. Thus, the color of purple flowers changed from red under 2,800 K fluorescent light (7°) to purple under white LED light (309°). In general, the purple color of flowers derives from the purple anthocyanin pigment accumulating in the vacuoles of the conical cells in the surface of the petals, or sometimes of the sepals^6^,^7^. Color changes observed in purple flowers may have some mechanism in common with alexandrite effect.

‘Psycho-physical’ reaction of the human eye to visible light spectrum is quantified with ‘color matching functions’ released from *Commission Internationale de l’Eclairage* (CIE). Color matching functions include ‘RGB color matching function’ based on standard red, green and blue colors, and ‘XYZ color matching function’ based on virtual X, Y and Z standard colors. Colors are plotted on various different diagrams derived from color matching functions. These diagrams were developed to intuitively discriminate colors according to three color factors, lightness (brightness), hue (e.g. red, green, blue) and saturation (vividness). This report also developed new color diagrams based on RGB color matching function, using triangular color vectors. One of these diagrams which was named ‘*round* diagram’ enables plotting all colors on a circle, according to their hues and saturations. This diagram helps us to better understand the colors of objects.

Colors of alexandrite and purple flowers were compared. Spectra of transmitted/reflected lights from these substances in different incident lights were estimated by measurements and calculations. These substances harbored quite different transmission/reflection spectra in different incident lights, consistent with alexandrite effect which they show in these incident lights. Lightness, hue and saturation of these substances were calculated with RGB color matching function, and plotted on *round* diagram. These analyses clarify the nature of interaction between light transmission/reflection of substances and psycho-physical reaction of the human eye, that result in alexandrite effect.

## Results

### Colorimetric explanation of the alexandrite effect in alexandrite stone

**Figure 1:**
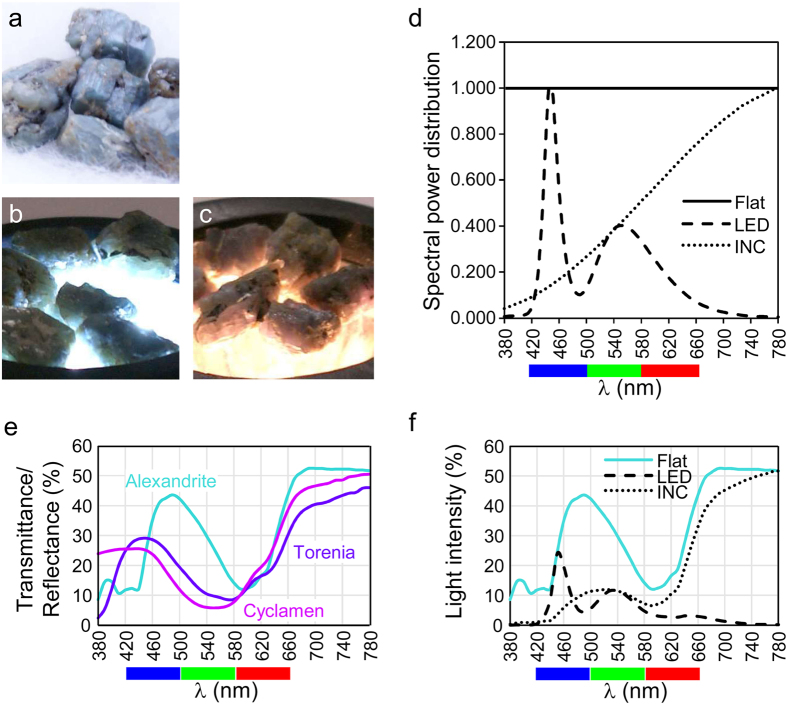
Transmittance of alexandrite and reflectance of purple flowers. Alexandrite gemstone lit by sunlight (**a**), LED light (**b**) or incandescent light (**c**). (**d**) Spectral power distribution (relative values) of hypothetical flat light (Flat), LED light (LED) and incandescent light (INC). Horizontal color bands at the bottom represent wave lengths of 420–500, 500–580 and 580–660 nm, which roughly correspond to blue, green and red. These bands are marked with these colors in the other figures as well. (**e**) Transmittance spectrum of alexandrite gemstone (emerald-green line) and reflectance spectra of torenia petal (blue line) and cyclamen petal (purple line). (**f**) Spectra of transmitted lights through alexandrite gemstone, illuminated with flat light, LED light or incandescent light.

Alexandrite effect is a well-known phenomenon, but there may not have been any precise colorimetric calculation of this effect. Figure 1(a–c) shows the colors of a low-quality alexandrite stone under sunlight, under slightly bluish white LED (light-emitting diode) light, and under rather orange incandescent light, respectively. The stones exhibiting the more pronounced colorimetric effects are usually the more precious ones. The stones in the figures exhibit different colors under different incident lights: cyanic under sunlight and LED light, and reddish under incandescent light.

Colors of transparent stones are calculated from the visible light spectra of the light sources and the transmittance spectra of the stones. The product of the light source spectrum and a stone’s transmittance spectrum yields the spectrum of the light coming out of the stone, then it is multiplied by the color matching function. Fig. 1d shows the measured spectra of the white LED and the orange incandescent light used in this study, together with a hypothetical (idealized) flat light source used to calculate the standard colors of objects. This flat light also mimics sunlight. With extreme simplification of the pattern of the RGB color matching function^8^,^9^, three continuous 80-nm ranges of visible light determine intensities of blue (420–500 nm), green (500–580 nm) and red (580–660 nm) colors. These approximate color ranges are shown beneath the wave lengths in the light spectra to aid intuitive understanding of color, which commonly results from complex patterns of light spectra. Thus, the white LED light is dominated by peaks in the blue and yellow (green plus red), whereas the incandescent light is dominated by strong red light, plus weaker greenish and bluish light.

The absorbance spectrum of alexandrite along the *b* axis was reproduced from an earlier publication^1^. For colorimetric analysis, the transmittance spectrum per 0.5-mm thickness of alexandrite stone was then calculated from the absorbance spectrum of alexandrite (Fig. 1e). This particular alexandrite gemstone has a wide cyanic transmittance peak around 500 nm and a weaker red transmittance slope at 620 nm and above. Figure 1f shows the spectra of transmitted light from alexandrite under flat light, under LED light, and under incandescent light. Under illumination by the flat light, the spectrum of the transmitted light is identical to the stone’s transmittance spectrum. The blue and green colors are enhanced compared with the original spectrum of incident light after passing through the alexandrite, giving the stone a cyanic color. Meanwhile, yellowish lights are absorbed more than the cyanic and reddish lights of the incandescent light source. The hue of the incandescent light after transmission through this alexandrite stone is not directly predictable from the spectrum pattern, although the overall brightness is clearly reduced after the light transmits through alexandrite.

### Alexandrite-like effect in purple flowers

**Figure 2:**
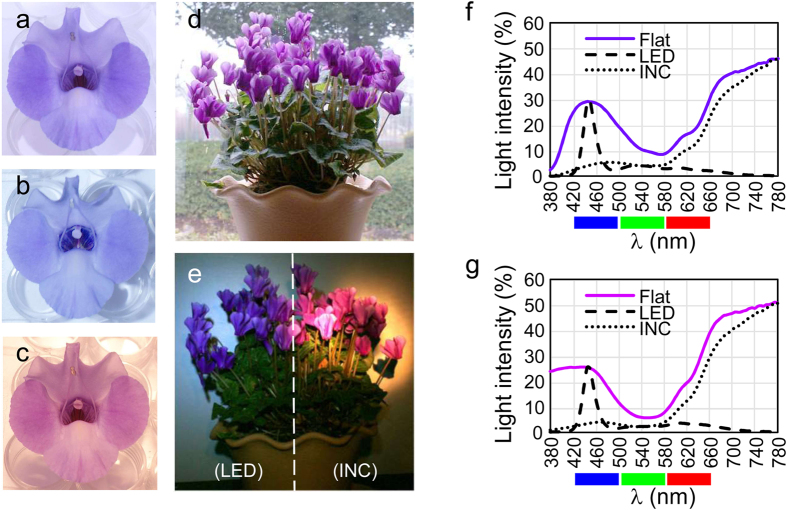
Colors of purple flowers. Torenia lit by sunlight (**a**), lit by LED light (**b**) and lit by incandescent light (**c**). Cyclamen lit by sunlight (**d**), lit by LED light (**e**), left) or lit by incandescent light (**e**), right). (**f**) Spectra of reflected light from torenia flower, lit by flat, LED or incandescent light. (**g**) Spectra of reflected light from cyclamen flower, lit by flat, LED or incandescent light.

A similar colorimetric phenomenon occurs in purple flowers. Consistent with this, the reflection spectra of visible light on the petals of purple torenia flowers and purple cyclamen flowers were similar to the transmission spectra through alexandrite stone (Fig. 1e). The petals of purple flowers had a moderate blue reflectance peak around 450 nm and a red reflectance slope at 620 nm and above. A mixture of blue and red reflected lights causes the flower to look purple. Figure 2(a–c) are photographs of the same purple ‘Summerwave Blue’ torenia flower, taken under sunlight, LED light or incandescent light, respectively. The flower is violet (blue-purple) in sunlight, blue under LED light and red under incandescent light. Similarly, the ‘Serenadia’ cyclamen flowers look purple in sunlight (Fig. 2d), violet under LED light, and red under incandescent light (Fig. 2e). The reflectance spectra of the torenia flower lit by flat light, LED light or incandescent light are shown in Fig. 2f. The torenia flower enhances the blue peak of the LED light and the red slope of the incandescent light, but it suppresses the yellow peak of the LED light and the greenish parts of the incandescent light. The same is true to the cyclamen flower (Fig. 2g). The enhancement/suppression pattern of specific parts of the incident light spectrum was even clearer in these purple flowers, than in the alexandrite gemstone. We will be able to refer to these flowers (or perhaps their anthocyanins) as ‘alexandrite-like’ materials, and as having ‘alexandrite-like’ properties.

### Calculation of colors with RGB vectors

Next, it is necessary to describe the characteristics of alexandrite and purple flowers with straightforward colorimetric calculations of the three factors of color: lightness, hue and saturation. Calculations of color adopt different formulae according to the choice of color system. For ease of understanding and other practical advantages, this report adopts the RGB color system and devise new color diagrams. Intensities of red, green and blue colors (*I*_R_, *I*_G_, *I*_B_) were calculated with the standardized RGB color-matching function used in our previous report^8^. Lightness (L^RGB^) (i.e. brightness) is simply calculated by the sum of *I*_R_, *I*_G_ and *I*_B_:


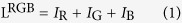


Chroma (vividness) values are inversely proportional to standard lightness. For example, a color with a full chroma value of 1.0 under some standard lightness, is only half vivid when chroma is calculated with a twice brighter standard lightness. This problem can be avoided if the lightness of each calculated color is standardized to 1. Standardized chroma, which can be derived from standardized color vectors, is called saturation. Standardized RGB intensities will be written as ρ, γ, and β where:


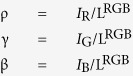


**Figure 3:**
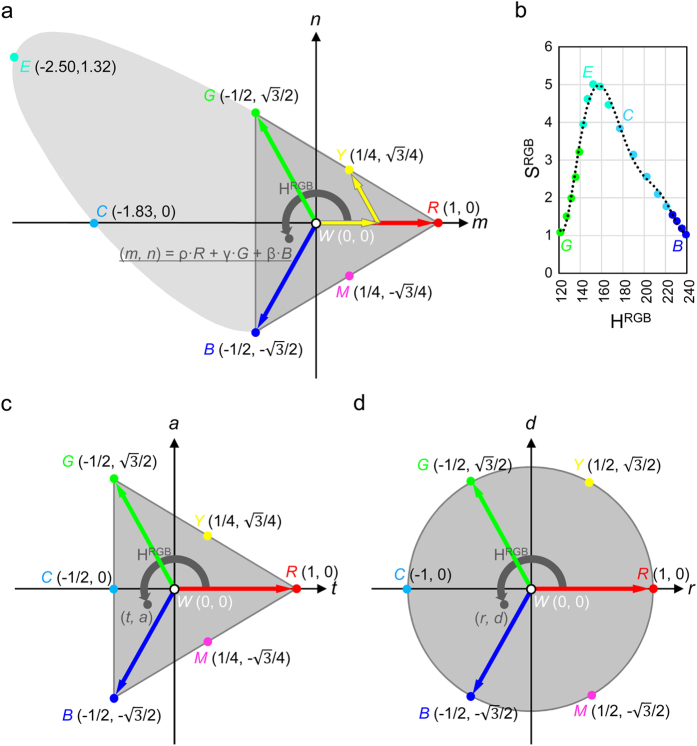
The *m-n* diagram, *triangle* diagram and *round* diagram. (**a**) *m-n* diagram. Coordinates of white (*W*), red (*R*), yellow (*Y*), green (*G*), emerald (*E*, λ = 510 nm), cyan (*C*), blue (*B*) and magenta (*M*) vectors are written in parentheses. The dark gray area represents the RGB triangle. Light gray area indicates cyanic color with negative ρ values. (**b**) S^RGB^ values shown as a function of H^RGB^ values. Color dots show representative values of greenish, emerald, cyanic and bluish colors. Dotted line represents the approximation (ASSCC). (**c**) *triangle* diagram with *t-a* coordinates. (**d**) *round* diagram with *r-d* coordinates.

Note that ρ, γ and β always sum to 1. After calculation of ρ, γ, and β, the colors are plotted on a two-dimensional *m*-*n* color plate (Fig. 3a). This *m-n* color plate corresponds to the ‘R + G + B = 1’ plate in RGB color space. Pure red, green and blue colors are expressed by the vectors 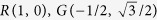 and 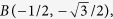 respectively. The other saturated colors are located on the lines/curve connecting between *R* and *G*, *G* and *B*, or *B* and *R*. For example, the unit yellow color vector 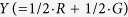 is located at the center of the line connecting between *R* and *G*, and the unit magenta color 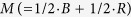 is located at the center of the line between *B* and *R*. Cyanic saturated colors (between *G* and *B*), such as emerald (*E*) and cyan (*C*) are located on the edge of the light-gray area in Fig. 3a. Saturated cyanic colors have negative ρ values. All standardized color vectors are expressed as follows:


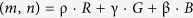


For calculation of saturation values, the smallest value among ρ, γ and β is important. When β is smallest, the vector contributing to saturation is:


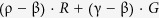


Then the remaining vector is colorless (i.e. white):


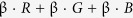


Saturation is expressed by the proportion of saturated vectors within the sum of all vectors:


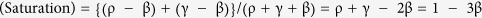


In general, saturation values calculated with the RGB vectors is:


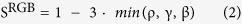


Here, the function ‘*min*’ represents the minimum value among the figures in the following parentheses. For yellowish colors (between red and green) and magenta colors (between blue and red), S^RGB^ ranges from 0 (white) to approximately 1 (saturated). Conversely, saturated cyanic colors (between green and blue) have S^RGB^ values far greater than 1. For example, the S^RGB^ value of an emerald color (λ = 510 nm) is as high as 5.01.

The third factor, hue, is expressed by the angle of each color vector. Color vector (*m*, *n*) is calculated as:


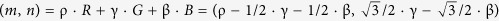


Hue is then calculated on the *m-n* color plate as:


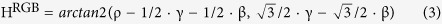


This formula for H^RGB^ is essentially the same as some of the previous reports^10^. Representative H^RGB^ values are 0°/360° (red), 60° (yellow), 120° (green), 180° (cyan), 240° (blue) and 300° (magenta). White does not have any H^RGB^ value. Thus lightness, saturation and hue are calculated with simple formulae (formulae 1, 2 and 3) based on the RGB color system.

### Triangular and rounded RGB diagrams with standardized saturation values

In addition to ease of calculation, an advantage of using the *m-n* color plate is that the hue angles of red, green and blue are evenly spaced on the diagram, in accordance with our perception of color. A disadvantage of the *m-n* color plate is distortion in the S^RGB^ values among colors (cyanic colors have much greater values than the others). The protruding curve of cyanic colors in the *m-n* diagram should be folded within RGB triangle.

Fig. 3b shows the relationship between the S^RGB^ and H^RGB^ values of cyanic colors. This irregular-shaped curve can be approximated with a polynomial function of the 6^th^ degree. This function is denoted ASSCC (approximated saturation of saturated cyanic colors):


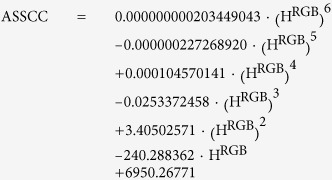


Nine significant digits are necessary to assure correct calculations of ASSCC. The standard deviation of the ratio between the actual saturation (S^RGB^) and the calculated saturation (ASSCC) of representative saturated cyanic colors was 0.05. Many of the cyanic colors found in nature would not be saturated. They have smaller S^RGB^ values than their hypothetical saturated counterparts. The S^RGB^ values of the measured colors can be standardized so that the saturations of saturated colors are approximately 1:


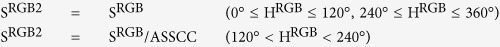


By dividing the *m-n* coordinates of cyanic colors by ASSCC, the protruding cyanic curve is folded within the RGB triangle. This color plot is denoted the *triangle* diagram. Coordinates are expressed as (*t*, *a*) (Fig. 3c):


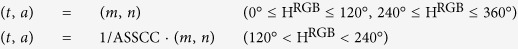


All saturated colors are placed on the three lines of the RGB triangle connecting between *R*, *G* and *B* in the *triangle* diagram. Alternatively, the color diagram can be readily rounded with trigonometric functions. This diagram is denoted the *round* diagram, with *r-d* coordinates (Fig. 3d):


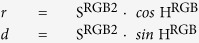


This *round* diagram perfectly fits our human perception of color. The colors - red, yellow, green, cyan, blue and magenta - are evenly placed along the margin of a circle, and the saturation values of saturated colors are approximately 1 (if not exactly 1) for any hue.

### Plotting colors of alexandrite-like materials on a *round* diagram

**Figure 4:**
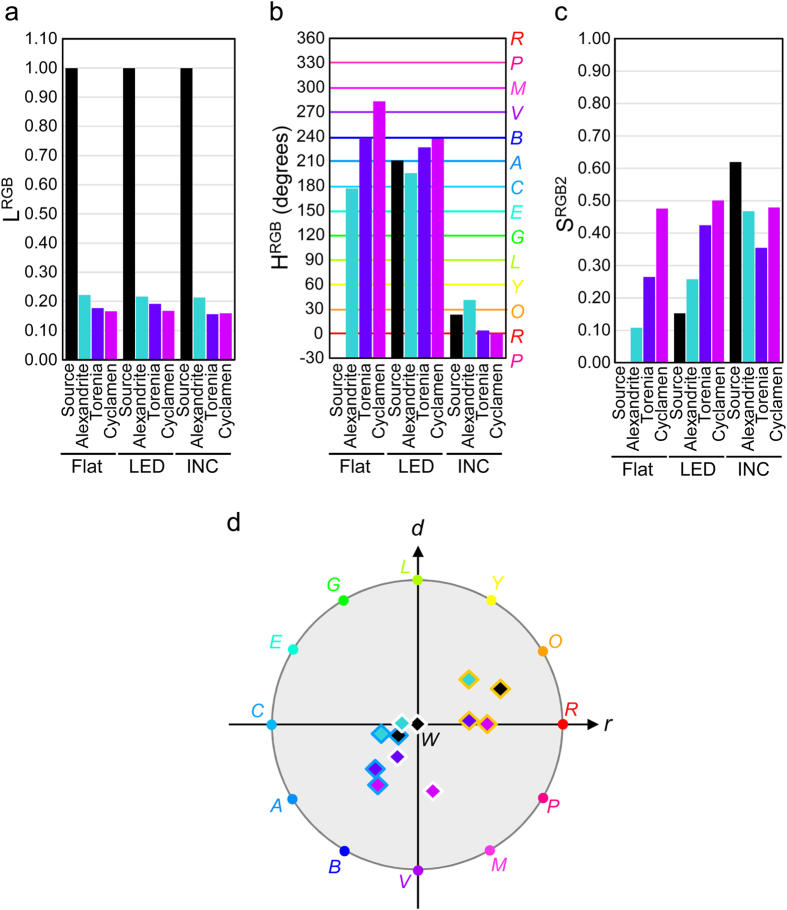
Colors of alexandrite-like materials. (**a**) Lightness of alexandrite-like materials illuminated with flat light (Flat), LED light (LED), or incandescent light (INC). Lightness of light sources (Source) are normalized to 1. (**b**) Hue of light sources and alexandrite-like materials. Color names are assigned every 30°: red (*R*), orange (*O*), yellow (*Y*), lawn (*L*), green (*G*), emerald (*E*), cyan (*C*), azure (*A*), blue (*B*), violet (*V*), magenta (*M*) and pink (*P*). (**c**) Saturation of light sources and alexandrite-like materials. (**d**) Colors of light sources and alexandrite-like materials plotted on the *round* diagram. Black, emerald, blue and purple diamonds correspond to light sources, alexandrite, torenia and cyclamen. Margin colors of diamonds show light sources illuminating the materials: flat light (white), LED light (cyanic blue), and incandescent light (orange). Coordinates of the 12 saturated colors (names as assigned in b) are show along the margin of the color circle.

Lightness, hue and saturation of the light source, alexandrite, torenia and cyclamen lit by flat light, LED light or incandescent light were estimated with L^RGB^, H^RGB^ and S^RGB2^ (Fig. 4a–c). Lightness was normalized with the intensities of each light source. Lightness of alexandrite and purple flowers were around 0.2 under any light. The LED light used here was light cyanic blue (H^RGB^ = 211°, S^RGB2^ = 0.15), and the incandescent light was a clear orange (H^RGB^ = 23°, S^RGB2^ = 0.62). The flat light was white (without H^RGB^ value, S^RGB2^ = 0.00). Alexandrite is cyan, torenia is blue, and cyclamen is purple in flat light. The color of alexandrite under flat light was lighter (the S^RGB2^ value was smaller) than those of the two flowers. Under flat light, the cyclamen had the darkest color among the three.

Colors of the alexandrite-like materials are similar to those of the LED light or incandescent light sources, although they are more or less different. Here, a minimum of 15° of hue difference was observed between the alexandrite-like materials and the light sources (LED light or incandescent light). This degree of difference in hue, accompanied by clear difference in lightness and saturation will be enough to make us feel that these objects have their own colors, unlike colorless objects showing just the same hue as the light source. Fig. 4d shows coordinates of alexandrite-like materials on a *round*-diagram. Alexandrite-like materials have a variety of colors according to the spectral quality of the light source.

## Discussion

The *round* diagram is similar to the popular HSV and HSL color spaces^10^. The HSV and HSL color spaces both divide hues into six components - red-yellow, yellow-green, green-cyan, cyan-blue, blue-magenta and magenta-red. A more correct hue calculation with arctangent function also seems to be adopted in some cases, in the same way as the H^RGB^ formula in this report. Looking at the area of actual color in the RGB coordinate system (Fig. 3a), the large area of cyanic colors protrudes from the RGB triangle. It would seem that the HSV and HSL systems neglect these protruding cyanic colors, generated by negative ‘ρ’ values. The solution suggested in this report is to treat this protrusion by ASSCC. With standardization of the saturation values of the cyanic colors with ASSCC, the *triangle* and *round* diagrams both contain all existing colors within the RGB triangle or color circle. Thus, for the first time, the *round* diagram offers a visible presentation that combines both the hue angle in line with the triangular RGB vectors and also the saturation value in proportion to the actual vividness of colors of any hue. The *round* diagram thus helps us to understand how colors differ from one another, on the basis of our natural color sense. The *r-d* coordinates of the *round* diagram are calculated with relatively simple formulae. To further improve intuitive understanding, we assigned names to the colors at 30° intervals (Fig. 4): red (R, 0°); orange (O, 30°); yellow (Y, 60°); lawn (L, 90°); green (G, 120°); emerald (E, 150°); cyan (C, 180°); azure (A, 210°); blue (B, 240°); violet (V, 270°); magenta (M, 300°) and pink (P, 330°).

Discrimination of similar colors is an important subject when discussing colors of objects. Then, what degree of hue or what percentage of lightness and saturation are enough for our eyes to discriminate colors? Millions of different colors can be generated on computer display^11^, but such similar colors would be difficult to discriminate for majority of people. ‘A Dictionary of Color’ and ‘Villalobos Colour Atlas’ contain 7,056 and 7,279 different color samples, respectively^12^. Munsell color system is a color space specifying all colors by hue, lightness and chroma. Colors are divided by 100 hues, 10 lightness levels, and around ten saturation levels. Judging from these figures, human eyes will be able to clearly discriminate around 10,000 different colors. This discrimination corresponds to 1% differences in hue (3.6°) and 10% differences in lightness and saturation.

Colors of alexandrite and purple flowers plotted on the *round* diagram (Fig. 4d) reveal the characteristics of alexandrite-like materials. Compared with the plots of light sources (three black diamonds), plots of alexandrite are evenly shifted to the left. This means that alexandrite adds a cyanic color vector to the light sources. Similarly, torenia adds a blue vector and cyclamen adds a violet vector. These added vectors correspond to the largest peaks of transmittance/reflectance spectra of these alexandrite-like materials (Fig. 1e). Unlike other substances, alexandrite-like materials have two transmittance/reflectance peaks at the cyan/blue and red wavelengths. This enables alexandrite-like materials to exhibit different hues under light sources of different spectral composition. Although the hues of alexandrite-like materials were similar to the LED light and incandescent light sources in this study, alexandrite-like materials can in principle exhibit hues that are completely different from the light source. Thus, in a showcase, an alexandrite gemstone can appear green on a reddish background if illuminated by a light source of the right spectral composition. Similarly, a flower in a café can appear blue under an orange light source.

The curvilinear and irregular shape of the light-response of the human eye is difficult to understand, and this sometimes results in mysterious color phenomenon. The mystery of the alexandrite effect is now better understood with the aid of a color diagram and a range of colorimetric functions.

## Methods

### Mineral and plant materials

A low-quality sample of the gemstone alexandrite was obtained via the internet. They emit red fluorescence under UV illumination, confirming that they are genuine alexandrite stones.

Violet torenia (*Torenia fournieri* Lind. ex Fourn. cultivar ‘Summerwave Blue’) and purple cyclamen (*Cyclamen persicum* Mill. cultivar ‘Serenadia’) are released by Suntory (Osaka, Japan). Torenia was grown in a naturally-lit greenhouse. This vigorous triploid cultivar sets relatively large flowers on creeping stems. Clones were propagated by directly inserting stems into the soil. Cyclamen was grown at the window. Cyclamen sets flowers continuously during winter and spring. The flowers of both torenia and cyclamen slightly change their color depending on growth conditions and the duration of flowering. Typical flowers were selected for analysis in this study.

### Spectral power distribution of light sources

The spectral power distributions of white (bluish) LED light and incandescent light were measured with MS-720 Spectroradiometer (EKO Instruments Co., Ltd., Tokyo, Japan). Spectra were proportionally standardized so that the maximum value is 1.

### Surface reflection spectra of flower petals

The reflection spectra of the adaxial (upper) surfaces of petals of torenia flowers and the adaxial (outer) surfaces of petals of cyclamen flowers were measured with a UV/VIS spectrophotometer UV-2450 (Shimadzu, Kyoto, Japan) equipped with a special attachment for reflection measurement. The spectra of transmitted/reflected light (*Spec*_λ_) under each light source were calculated from the transmission/reflection spectra (*R*_λ_) of gemstones and flowers and the light-source spectra (*L*_λ_):


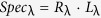


### Photographs

All photographs were taken with a digital single-lens reflex camera DSLR-A300 (Sony, Tokyo, Japan). Response curves for the RGB sensors of this camera were not available when inquiry was made to the manufacturer. Response curves of most cameras seem to differ greatly from the shape of the RGB color matching function. Thus it is impossible to perfectly regenerate colors, especially purple and violet colors which are red/blue mixtures. Colors may also be altered when images are re-formatted and printed. The colors in the images presented in this report are as close as possible to the original subjects.

### Calculation of RGB intensities

Standardized RGB color matching functions *F*_R_, *F*_G_ and *F*_B_ are provided as one of the supplementary materials in our previous report^8^. The RGB intensities (*I*_R_, *I*_G_ and *I*_B_) of a given light spectrum (*Spec*_λ_) were calculated as follows:


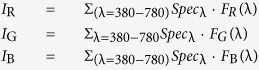


Transmission (*Tra*) of alexandrite was calculated from absorbance (*Abs*) as:


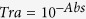


All calculations including ASSCC were performed by Microsoft’s Excel software with default settings.

## Additional Information

**How to cite this article**: Kasajima, I. Alexandrite-like effect in purple flowers analyzed with newly devised round RGB diagram. *Sci. Rep.* **6**, 29630; doi: 10.1038/srep29630 (2016).
